# Supplementary material for: One-Step Synthesis of CuxOy/TiO2 Photocatalysts by Laser Pyrolysis for Selective Ethylene Production from Propionic Acid Degradation
Source: Nanomaterials (Basel). 2023 Feb 21;13(5):792. doi: 10.3390/nano13050792 (PMC10005428; doi:10.3390/nano13050792)
Supplement: Supplementary file 1 [file nanomaterials-13-00792-s001.zip › nanomaterials-2211518-supplementary.pdf]

Supplementary Materials

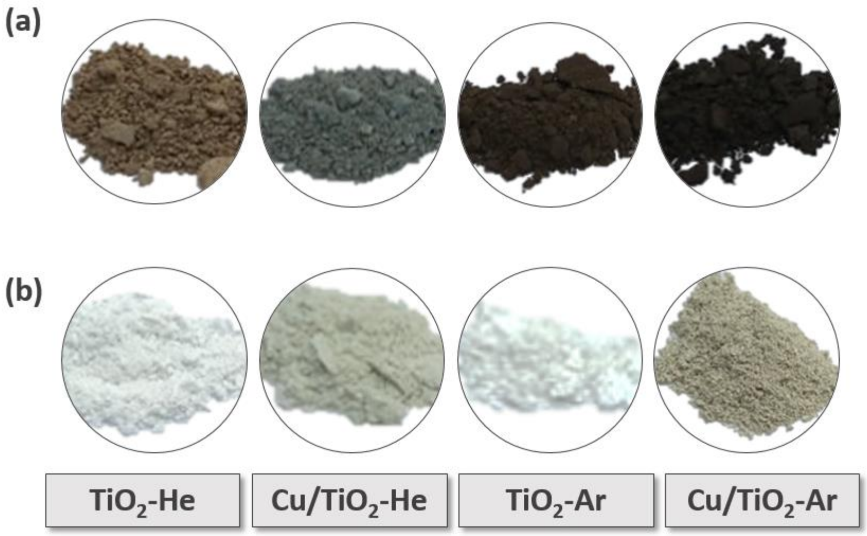

Figure S1. Images of TiO_2 and Cu/TiO_2 samples before (**a**) and (**b**) after annealing.

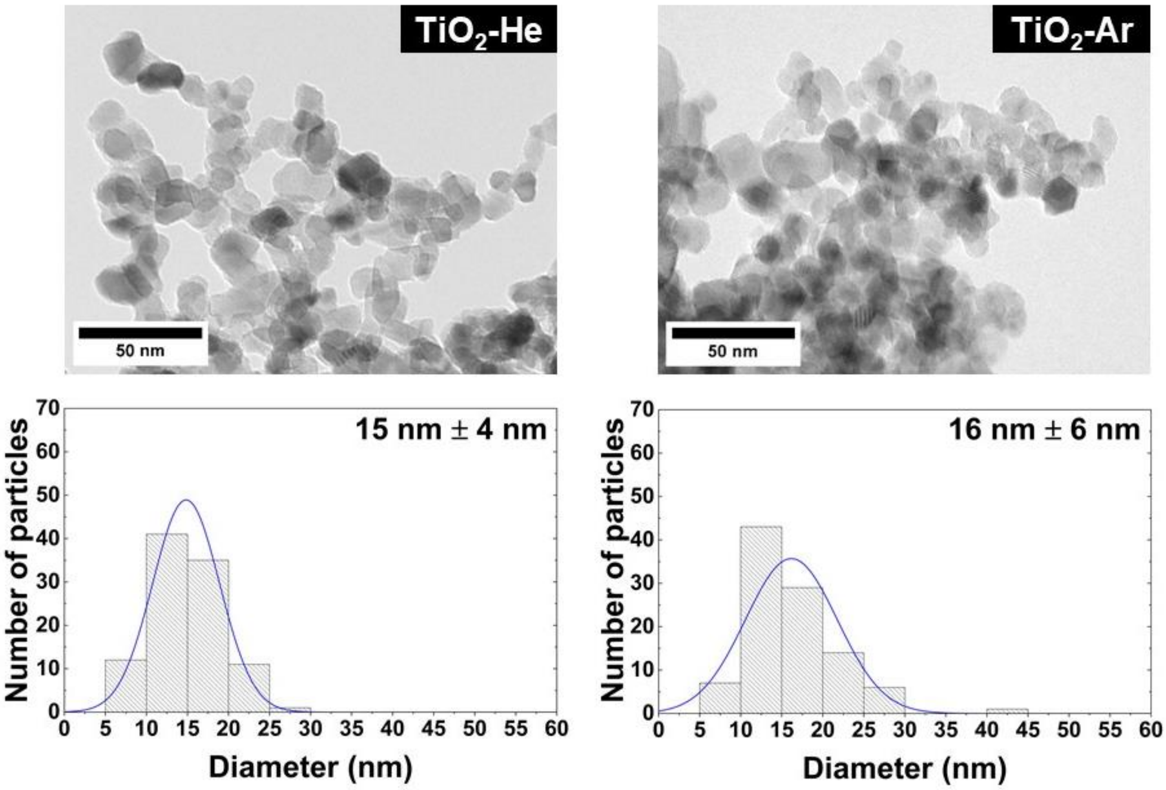

Figure S2. TEM images and associated histograms of size distribution of TiO_2 powders. Diameter measurements were performed on 100 particles with ImageJ 1.53t software.

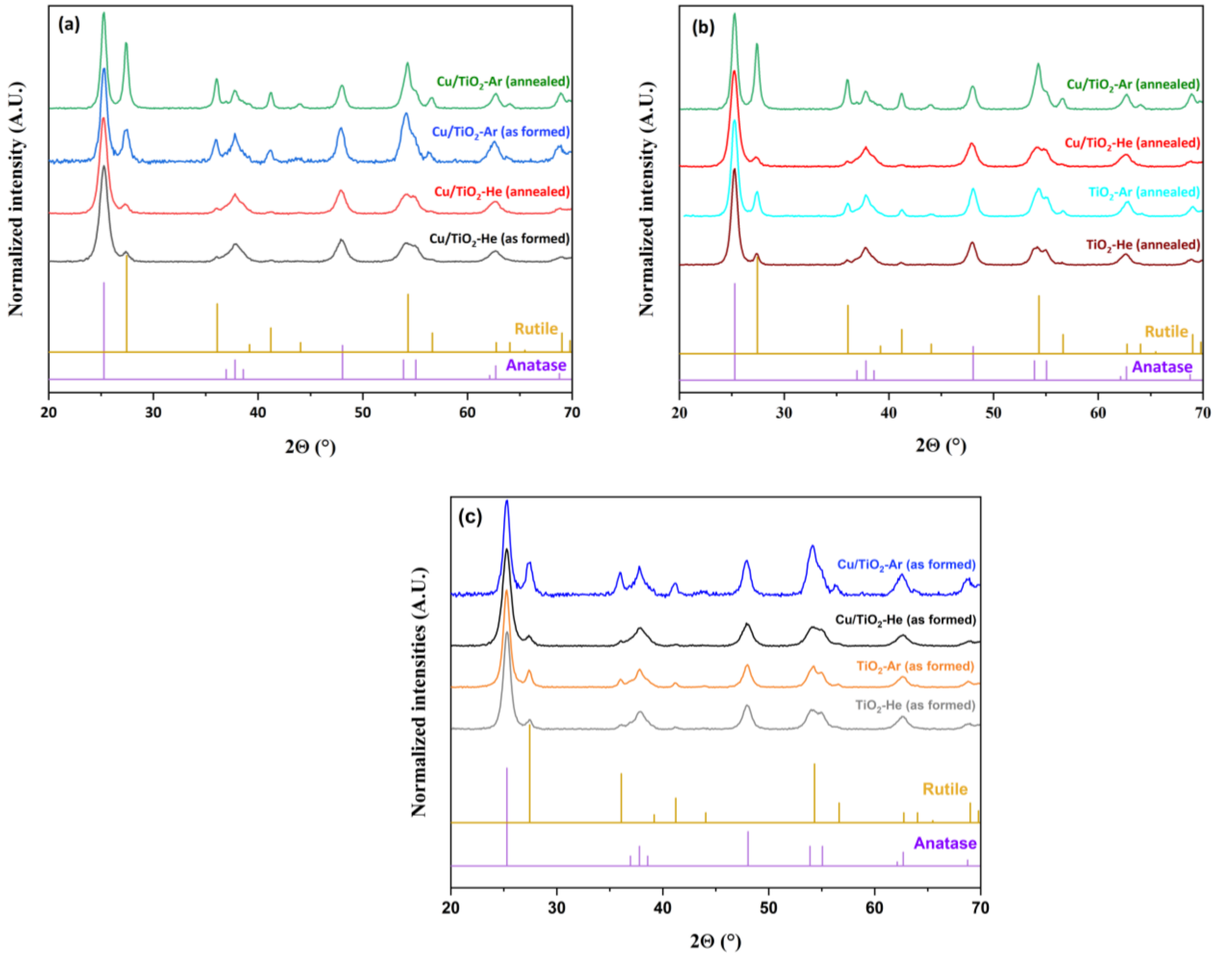

Figure S3. XRD patterns of Cu/TiO_2 samples before and after annealing (**a**); TiO_2 and associated Cu/TiO_2 samples after annealing (**b**); XRD patterns of TiO_2 and Cu/TiO_2 as formed (**c**).

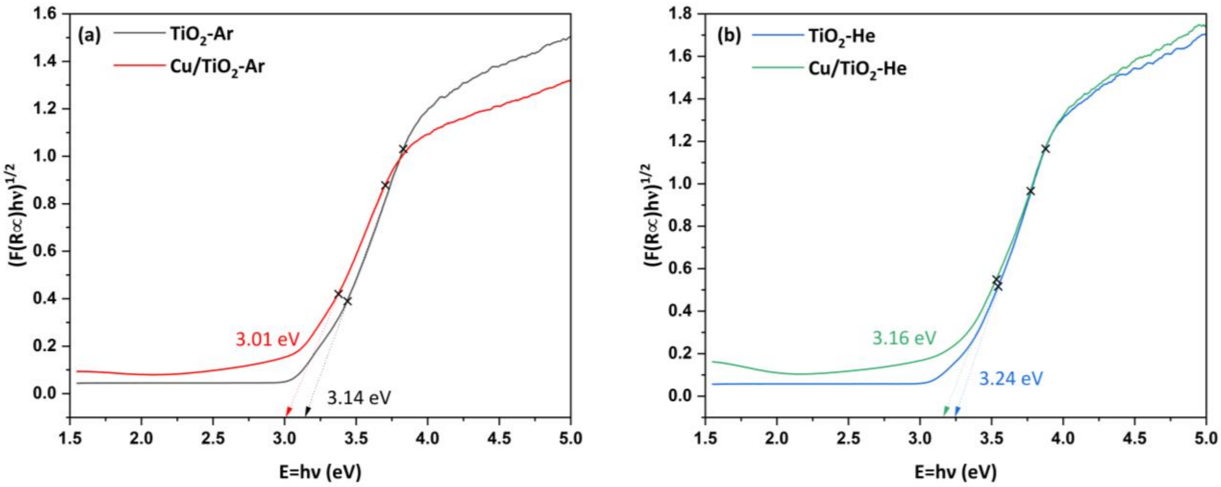

Figure S4. Optical gap determination from UV-vis diffuse reflectance spectra using modified Kubelka-Munk function [1] considering indirect gap of TiO_2.

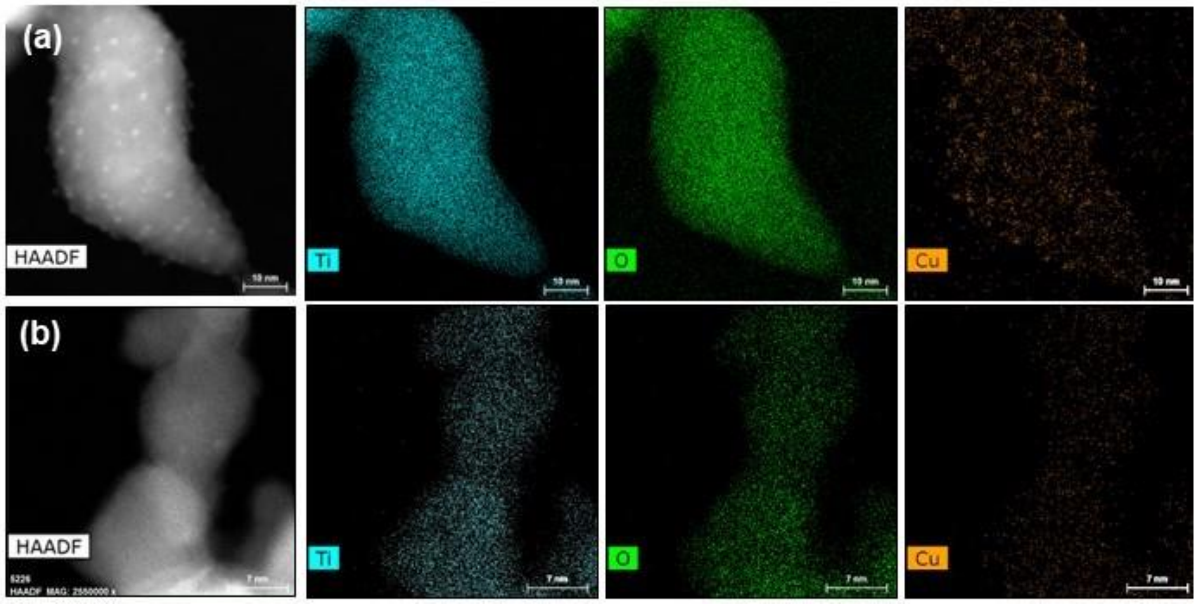

Figure S5. STEM-HAADF images and Ti, O, Cu elemental maps performed on Cu/TiO_2-Ar (**a**) and Cu/TiO_2-He (**b**).

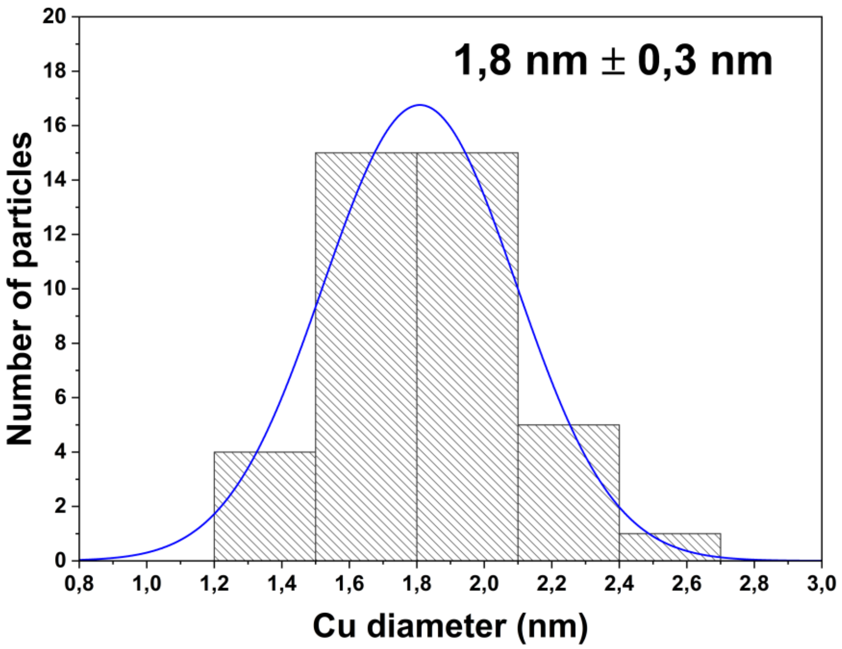

Figure S6. Histograms of size distribution of Cu nanoparticles in Cu/TiO_2-Ar. Diameter measures were realized on 40 particles with ImageJ software.

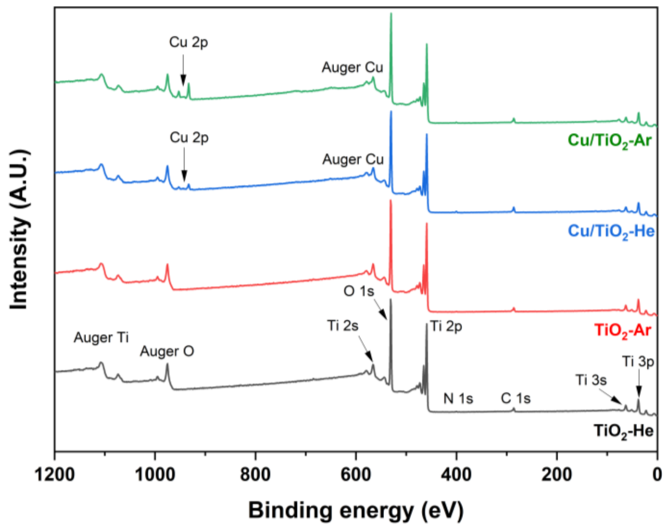

Figure S7. XPS survey spectra of synthetized TiO_2 and Cu/TiO_2.

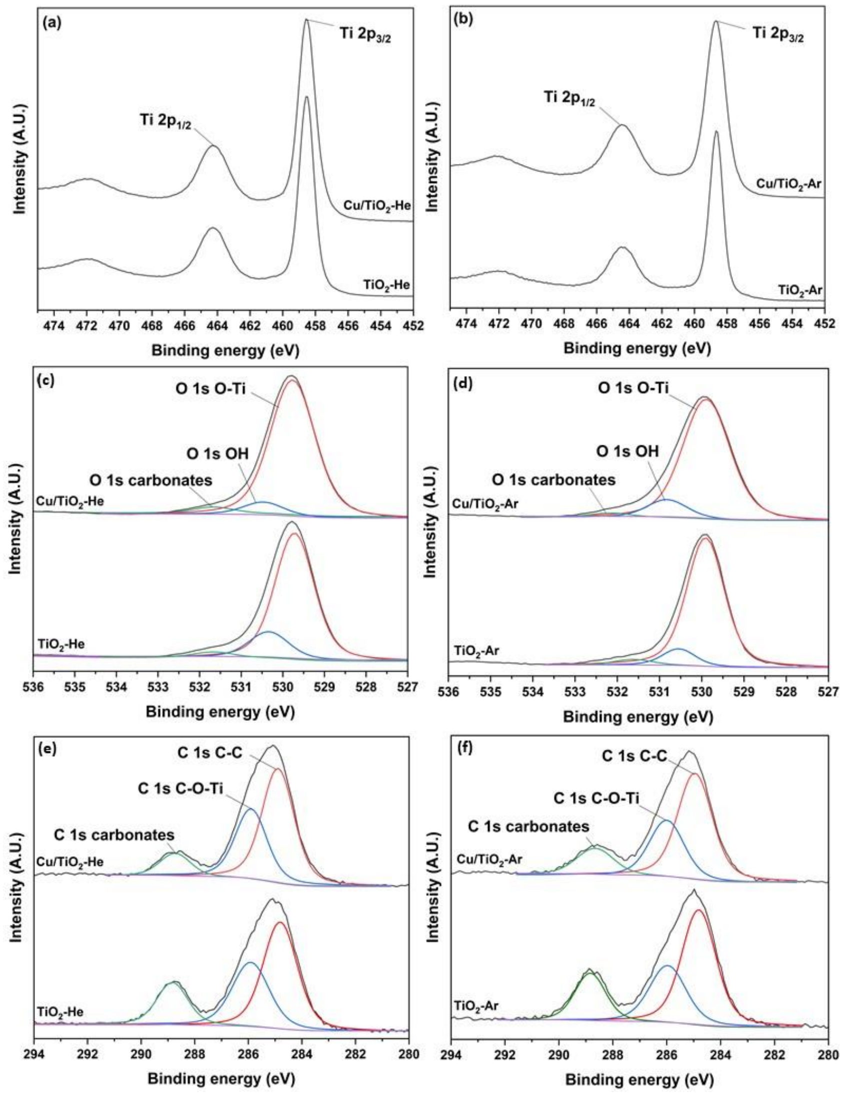

Figure S8. Ti 2p (**a,b**), O 1s (**c,d**) and C 1s (**e,f**) core-level spectra of synthetized TiO_2 and Cu/TiO_2.

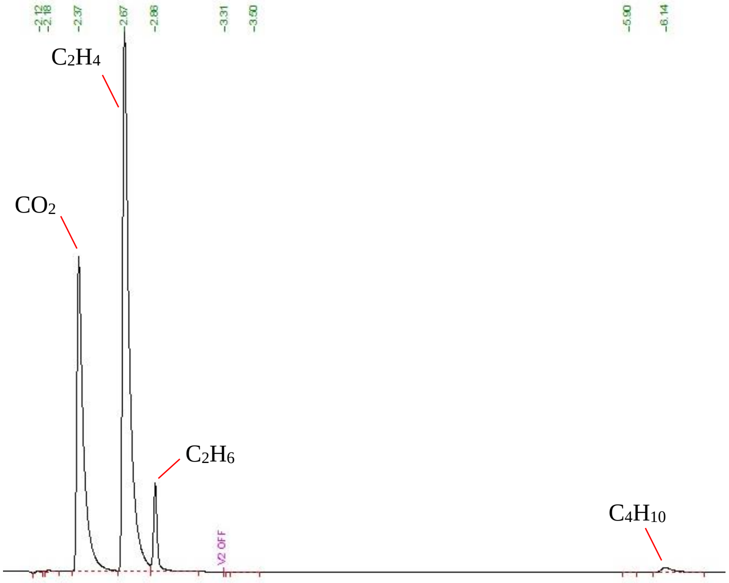

Figure S9. FID (Flame Ionization Detector) gas chromatogram of after 910 minutes of irradiation from PA (1 vol%) photo-decarboxylation with Cu/TiO_2-Ar. Gas identifications were made with calibration cylinders of CO_2 alone (1000 ppm in nitrogen), C_2H_6 alone (500 ppm in nitrogen) and a mixture of C_2H_6 and C_2H_4 (10 ppm in nitrogen). Retention times (min) are in green.

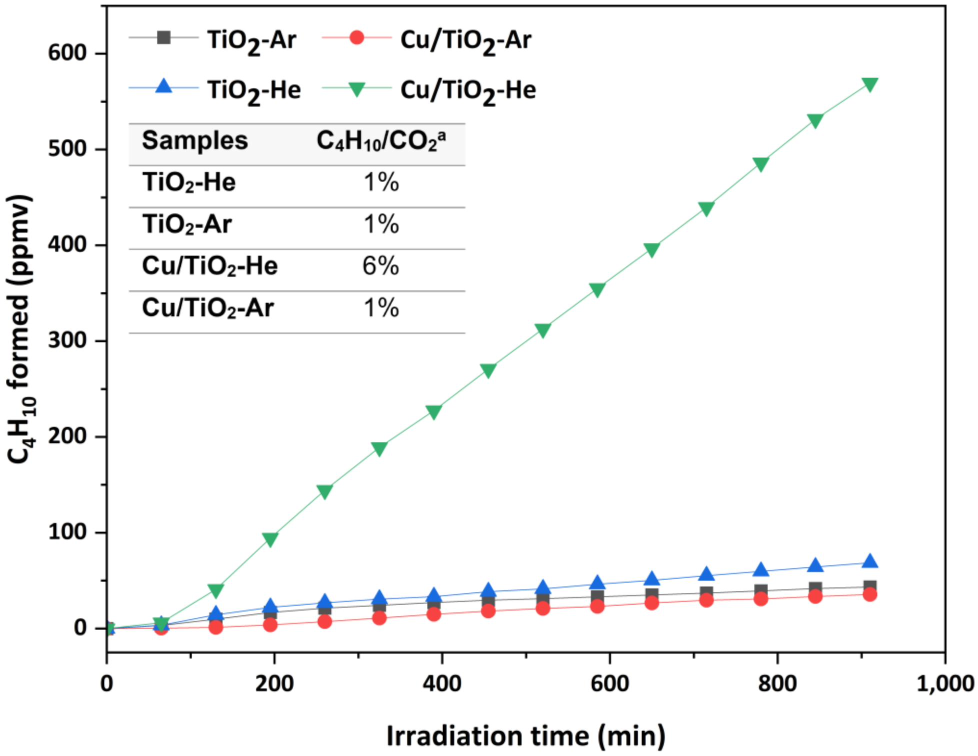

Figure S10. C_4H_{10} formation from PA (1 vol%) photo-decarboxylation under UVA light. C_4H_{10}/CO_2 ratios are calculated at 910 min of irradiation.

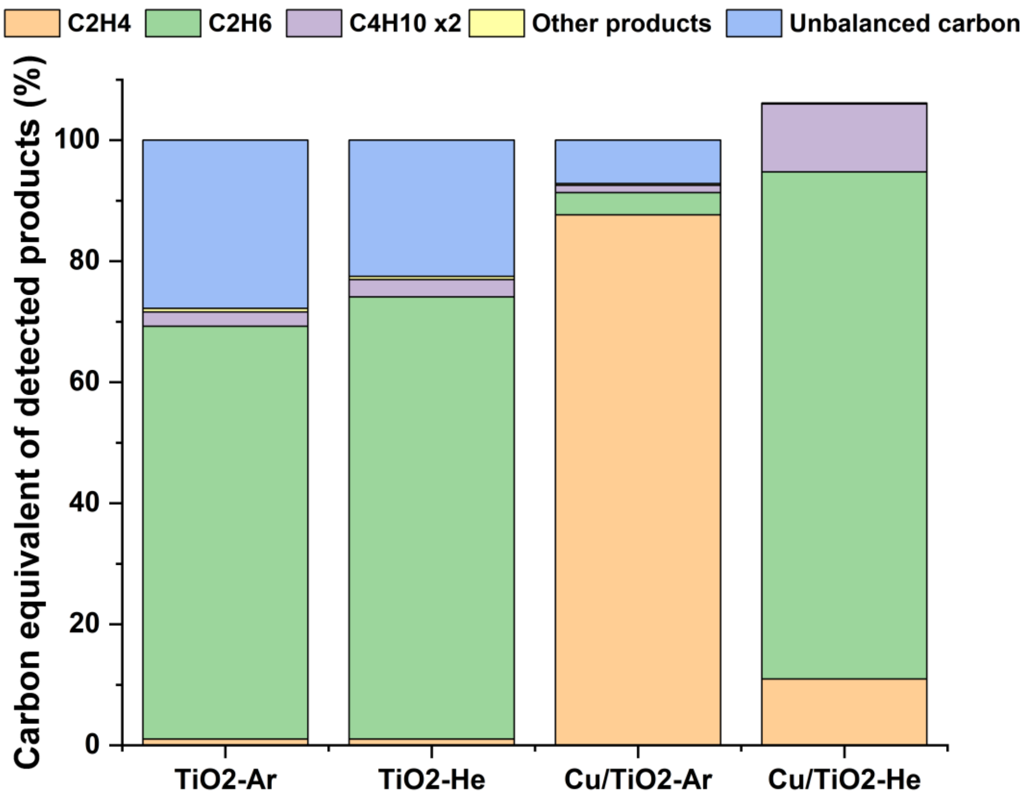

Figure S11. Carbon equivalent of each detected product by GC after 910 min of irradiation of PA (1 vol%). Calculations are made considering 1 CO_2 molecule for 1 C2 product (C_2H_4, C_2H_6, acetaldehyde) and 2 CO_2 molecules for C4 product (C_4H_{10}).

References

1. Simmons, E.L. Diffuse reflectance spectroscopy: a comparison of the theories. Appl. Opt. 1975, 14, 1380–1386, https://doi.org/10.1364/ao.14.001380.
